# Phage therapy: a promising approach for *Staphylococcus aureus* diabetic foot infections

**DOI:** 10.1128/jvi.00458-25

**Published:** 2025-05-14

**Authors:** Lucile Plumet, Chloé Magnan, Denis Costechareyre, Albert Sotto, Jean-Philippe Lavigne, Virginie Molle

**Affiliations:** 1 VBIC, INSERM U1047, University of Montpellier 27037, Montpellier, France; 2 VBIC, INSERM U1047, University of Montpellier, Department of Microbiology and Hospital Hygiene, CHU Nîmes, Nîmes, France; 3 Greenphage, Cap Alpha, Clapiers, France; 4 VBIC, INSERM U1047, University of Montpellier, Department of Infectious Diseases, CHU Nîmes, Nîmes, France; New York University Department of Microbiology, New York, New York, USA

**Keywords:** diabetic foot infections, *Staphylococcus aureus*, phage therapy, bacteriophages, animal models, clinical cases, clinical trials

## Abstract

Diabetic foot infections (DFIs), predominantly caused by *Staphylococcus aureus*, pose a significant healthcare challenge with severe consequences, including amputation. Phage therapy, which utilizes bacteriophages to specifically target bacterial pathogens, has emerged as a promising alternative to conventional antibiotic treatments. This review evaluates the efficacy of phage therapy as a complementary treatment for DFIs caused by *S. aureus*, synthesizing evidence from preclinical and clinical studies while addressing the limitations and challenges associated with current research. The analysis highlights promising results from diabetic animal models, demonstrating effective bacterial load reduction and improved wound healing. Clinical case reports and series further underline significant improvements in infection management and ulcer healing, with no major adverse effects reported. Ongoing clinical trials are also discussed, offering insights into the study parameters evaluating phage therapy potential efficacy and safety for *S. aureus*-related DFIs. While the collected data highlight the potential of phage therapy as a valuable complement to traditional antibiotic treatments, particularly in managing antibiotic-resistant infections, further research is essential to address existing limitations, including gaps in long-term efficacy data and challenges in standardization. With continued investigation, phage therapy holds significant potential to alleviate the healthcare burden of DFIs and improve patient outcomes.

## INTRODUCTION

Diabetic foot infections (DFIs) represent a significant and growing challenge in modern healthcare, particularly due to their association with high morbidity, increased healthcare costs, and the risk of amputation (1, 2). *Staphylococcus aureus*, identified in approximately 50% of DFIs, is frequently associated with severe and difficult-to-treat cases. This bacterium is a major contributor to the complications associated with DFIs, including the development of chronic, non-healing wounds and osteomyelitis. In severe cases, the chronic nature and persistence of *S. aureus* infections can necessitate limb amputation, significantly impacting the patient’s quality of life and increasing the burden on healthcare systems (3, 4). While current antibiotic therapies are essential, they face limitations. The presence of methicillin-resistant *S. aureus* (MRSA) or multidrug-resistant (MDR) strains, combined with the reduced efficacy of antibiotics in chronic biofilm-associated infections, exacerbates treatment challenges, leading to higher rates of re-infection and increased antibiotic use (5).

Phage therapy, which uses bacteriophages (or phages) to target and eliminate specific bacterial pathogens, is emerging as a promising alternative to traditional antibiotic treatments. Phages offer several advantages: they have high specificity for their pathogen bacterial hosts, thus preserving the beneficial microbiota, and they can replicate at the site of infection, thereby increasing their efficacy over time (6). Notably, staphylococcal phages, especially those of the *Silviavirus* and *Kayvirus* genera, combine a broad host range across clinical isolates with strong biofilm-disrupting abilities, making them particularly well-suited for treating infections like DFIs, where biofilms represent a major therapeutic challenge (7–9).

While the efficacy of phage therapy has already been demonstrated in various animal models, it is crucial to focus on models that specifically mimic the pathophysiology of DFIs to better evaluate their relevance and effectiveness in this context. This enables a deeper understanding of their effects in a living organism and a more accurate assessment of their effectiveness (10–12). These models provide valuable insights that bridge the gap between laboratory studies and clinical applications, ensuring that therapies are both safe and effective before being tested in human patients (13). This review aims to explore the efficacy and limitations of phage therapy in the context of DFIs caused by *S. aureus*, examining both preclinical and clinical evidence to assess its potential as a game-changing treatment. We performed a comprehensive literature search across PubMed (NLM Database-MEDLINE (OvidSP) and ClinicalTrials.gov, including terms such as “diabetic foot infections,” “diabetic foot osteomyelitis/osteitis,” “*Staphylococcus aureus*,” and “phage therapy,” along with “animal model” and “case report/series.” The results are organized in Table 1 for studies involving diabetic animal models, in Table 2 for clinical case reports, and in Table 3 for clinical trials.

## EVALUATING PHAGE THERAPY IN DIABETIC ANIMAL MODELS SIMULATING *S. AUREUS* DFIs

Diabetic animal models are essential for assessing the potential of phage therapy against *S. aureus*-caused DFIs. Rodents, especially mice and rats, are commonly used due to their size, affordability, and genetic modifiability, allowing detailed study of infection treatments and wound healing. Larger animals, such as rabbits and pigs, although less frequently used due to cost and ethical concerns, provide a closer physiological match to humans. Pigs have skin structures resembling human skin, making them valuable for extensive wound experiments (14, 15). Together, these models help to simulate diabetic conditions, enabling precise studies on diabetes induction, infection progression, and phage therapy interaction with or without antibiotics, which are critical for translating research findings into therapeutic applications (13).

To study phage therapy against *S. aureus* in DFIs, diabetes is commonly induced in animal models using chemical agents like streptozotocin (STZ) and alloxan (Table 1). STZ induces selective DNA alkylation in pancreatic β-cells, which ultimately leads to their destruction. This process results in insulin deficiency and hyperglycemia, making STZ an effective agent for modelling Type 1 diabetes. STZ can be administered as a single high dose to induce acute diabetes or as smaller, repeated doses to gradually develop the disease, thereby representing different stages of the diabetic pathology (14, 16). Alloxan induces diabetes by generating reactive oxygen species that lead to β-cell necrosis. Although cost-effective, it is less commonly used due to instability and potential severe side effects. After diabetes induction, studies rely on two strategies: either direct bacterial injection or generating surgical wounds for subsequent inoculation (Table 1). Direct injection, often used in smaller animals like mice, introduces *S. aureus* intraperitoneally (17) or in specific sites such as the hindpaw (18, 19) to allow precise infection control. Alternatively, surgical wounds used in various species (e.g., mice, rats, rabbits, and pigs) tend to mimic diabetic patient wounds by varying wound sizes to reflect clinical scenarios. Afterward, *S. aureus* is applied, simulating infection conditions with doses from 10^6^ to 10^9^ colony-forming units (CFU).

After inducing *S. aureus* infection, phage therapy treatment is applied, with timing, dosage, and method varying across studies. Phage doses typically range from 10^5^ to 10^9^ plaque-forming units (PFU), administered either as a single dose or multiple doses over several days (Table 1). In some studies, phages are administered within a 30-min timeframe post-infection to prevent the establishment of the infection, demonstrating high efficacy in diabetic BALB/c mice (17–20). Other studies administer phages several days post-infection to evaluate their effectiveness on established infections, with the first treatment typically administered 2–4 days after infection and the final treatment up to 8 days later. This method demonstrated success in reducing bacterial load and promoting wound healing (21, 22). Interestingly, the specific pathophysiological context of DFIs may enhance the effectiveness of phage therapy. Their localized and poorly vascularized nature could allow phages to persist longer and act more effectively at the infection site, with limited exposure to systemic immune clearance. This local environment may partly explain the encouraging therapeutic outcomes observed in diabetic animal models (23, 24).

Moreover, phages can be used as monotherapy or in cocktails. Ghanaim et al. (25) found that a phage cocktail targeting a polymicrobial infection of *S. aureus*, *Pseudomonas aeruginosa*, *Klebsiella variicola*, and others significantly improved wound healing over ceftriaxone, enhancing bacterial reduction, collagen deposition, and tissue structure (25). In fact, phage therapy is often combined with antibiotics to explore potential synergies. However, studies suggest mixed outcomes; while one study showed that a combination of a phage cocktail with vancomycin achieved greater bacterial load reduction in diabetic mice than either treatment alone (21), another found no added benefit from combining phages with linezolid for *S. aureus* infections, highlighting that not all phage-antibiotic combinations are synergistic (18). These findings highlight the importance of careful selection in phage-antibiotic protocols, as combinations do not always yield beneficial outcomes, emphasizing the need to test phages both alone and in combination with antibiotics.

An additional advantage of phage therapy is its ability to target bacterial infections without compromising the host natural microbiota. Huon et al. (26) demonstrated that phage therapy not only reduces bacterial load and promotes wound healing but also preserves gut microbiota integrity, unlike antibiotics, which can disrupt this balance (26). Furthermore, Ghanaim et al. (25) highlighted the role of phages in modulating immune responses, offering benefits in bacterial reduction, microbiota preservation, and inflammation control. These findings suggest that phages provide therapeutic benefits beyond merely targeting bacterial infections; they also help minimize side effects on the host by preserving microbiota balance and reducing inflammation, making phage therapy a promising approach with broad therapeutic potential.

While existing models for evaluating phage therapy against *S. aureus* in diabetic conditions provide valuable insights, they are primarily based on agents like STZ or alloxan to simulate Type 1 diabetes. This approach does not accurately reflect the physiology of Type 2 diabetes, which is more prevalent among patients with DFIs (27). Although models for Type 2 diabetes have been developed, they are not applied in phage therapy research and often fail to encompass the complex dynamics of human DFIs. Critical factors, such as the impact of comorbidities like neuropathy and vascular complications on infection progression and wound healing, are frequently overlooked. Another notable limitation is the nature of the wounds studied, as they are acute wounds, while chronic wounds, which persist for over 4 weeks, are more representative of clinical DFIs. These physiological and methodological gaps limit the translation of phage therapy research findings to clinical settings. Moreover, variability in experimental parameters, such as the *S. aureus* inoculum, phage dosage, and administration methods, reduces the comparability of results across studies. Moreover, the short duration of most animal studies limits the evaluation of long-term effects, including phage resistance development and microbiota alterations. These challenges highlight the need for more standardized and clinically relevant models to fully realize the potential of phage therapy and support the development of effective, tailored treatments for DFIs.

**TABLE 1:** Efficacy of phage therapy against *S. aureus* infections mimicking DFIs in diabetic animal models^*a*^

Animal model	Diabetes induction method	Infection details	Phage therapy protocol	Combination therapy	Outcomes	Ref.
Small animal model						
BALB/c female mice	Two intraperitoneal injections of 150-mg/kg STZ at 48 h apart	Single injection of 10^7^ CFU into the hindpaw (*S. aureus* Hocil17, MSSA)	Single injection of 10^8^ PFU into the hindpaw (phage PP1493, PP1815, and PP1957 in cocktail), 30 min post-infection	Single intraperitoneal injection of linezolid 25 mg/kg	The phage cocktail was effective in both non-diabetic and diabetic mice. Linezolid demonstrated similar efficacy to the phage cocktail in non-diabetic mice but was less effective in diabetic mice. No synergistic effect was observed with the combination therapy.	(18)
4- to 6-week-old BALB/c female mice	Two intraperitoneal injections of 150-mg/kg alloxan monohydrate at 48 h apart (mice fasted overnight)	Single injection of 10^6^ CFU into the hindpaw (*S. aureus* ATCC 43300, MRSA)	Single local administration of 10^8^ PFU into the hindpaw (phage MR-10), 30 min post-infection	Single oral administration of linezolid 25 mg/kg	Phage treatment was highly effective and comparable to linezolid. Combination therapy resolved hindpaw infection more effectively.	(19)
4- to 6-week-old BALB/c female mice	Two intraperitoneal injections of 150-mg/kg alloxan hydrate at 48 h apart (mice fasted overnight)	Single local injection of 10^8^ CFU into an open wound (*S. aureus* ATCC 43300, MRSA)	Single local administration of 10^9^ PFU into the wound (phage MR-5, MR-10, single, in cocktail, or entrap in liposomes), 30 min post-infection	Two intraperitoneal injections of clarithromycin 10 mg/kg in 12-h intervals	The phage cocktail was highly active and more effective than monophage therapy. Liposome-entrapped phage cocktail improved persistence and infection resolution compared to free phage cocktail or clarithromycin.	(20)
10-week-old RjOrl/SWISS female mice	Intraperitoneal injection of 170-mg/kg STZ	Single local administration of 10^8^ CFU on a wound (*S. aureus* NSA1385, MSSA)	Single local administration of 10^5^ PFU over the wound (phage PN1815, PN1957 in cocktail), 48 h post-infection	Oral administration of amoxicillin-clavulanic acid 60 mg a day for 5 days	Phage therapy reduced bacterial load and improved wound healing without disrupting intestinal microbiota. Antibiotics improved wounds more slowly and disrupted microbiota diversity. Combined therapy was not more effective than phage alone.	(26)
8-week-old BALB/c female mice	Intraperitoneal injection of 50 mg/kg of STZ for 5 days, after 4 h of fasting	Single local administration of 5.10^6^ CFU into wounds (*S. aureus* 63–2498, MDR)	Local administrations of 8.10^7^ PFU to wounds (phage cocktail AB-SA01), on days 3, 5, and 7 post-infection	Two intraperitoneal injections of vancomycin 150 mg/kg per day for 5 days	The phage cocktail reduced bacterial load as effectively or better than vancomycin, with similar wound healing outcomes.	(21)
6-week-old BALB/c female mice	Intraperitoneal injection of 60–180 mg/kg of STZ for 3 days	Single intraperitoneal injection of 2.10^8^ CFU (*S. aureus* RCS21)	Single intraperitoneal injection of 10^9^ PFU (phage GRCS), 30 min post-infection	Single intraperitoneal injection of oxacillin 20 mg/kg per day for 5 days	A single phage dose protected diabetic and non-diabetic mice from lethal bacteremia. Phage therapy and oxacillin both significantly decreased bacterial load in both mouse models, even when phage treatment was delayed by up to 4 h.	(17)
8- to 10-week-old male Sprague-Dawley rats	Intraperitoneal injection of 65 mg/kg of STZ	Single local administration of 2.10^8^ CFU into a wound (DFI isolates of *S. aureus, P. aeruginosa, K. variicola, E. coli, Proteus mirabilis*)	Local administrations of 10^9^ PFU to the wound (phage S2, Ps&, K4, C3, Pr2 in cocktail), 48 h post-infection for 5 days	Local administration of 2% ceftriaxone, 2 days post-infection for 5 days	The phage cocktail proved more effective than ceftriaxone in all wound healing measures, including wound size, bacterial load, collagen deposition, and histomorphic appearance in diabetic infected wounds.	(25)
8- to 10-week-old male Wister rats	Intraperitoneal injection of 40 mg/kg of STZ	Single injection of 2.10^7^ CFU into a wound (one clinical wound isolate of *S. aureus*)	Local administrations into the wound after debridement (phage F44/10, F125/10), every 4 h on day 4 post-infection, and every 12 h from day 5 to day 8 post-infection		Phage cocktail effectively decreased bacterial load and improved wound healing.	(22)
6-month-old male Wister rats	Subcutaneous injection of 80 mg/kg of alloxan hydrate, after 12 h of fasting	Single local administration of 10^9^ CFU into a wound (DFI isolate of *S. aureus* CSV-36, MRSA)	Single local administration of 3.10^9^ PFU (phage SH-56), 4 days post-infection	Single administration of clindamycin 8 mg/kg	The phage-treated group showed significant reduction in infection, faster epithelization, and improved wound contraction compared to the antibiotic-treated diabetic rats and the control group.	(28)
Large animal model						
2- to 3-month-old male rabbits	One to two subcutaneous injections of 80 mg/kg of alloxan hydrate, after 12 h of fasting	Single local administration of 10^7^ CFU into a wound (DFI isolate of *S. aureus* CSV-36, MRSA)	Single local administration of 3.10^9^ PFU (phage SH-56), 48 h post-infection	Single administration of clindamycin 8 mg/kg	The phage-treated group showed significant decreases in infection, epithelization period, and wound contraction compared to antibiotic-treated and control groups.	(29)
Female Yorkshire pigs	Injection of 150-mg/kg STZ via ear vein catheter, after 12 h of fasting	Single local administration of 2.10^6^ CFU into wounds (clinical isolate of *S. aureus*)	Local administrations into the wound after debridement (phage F44/10, F125/10), every 4 h for 24 h on day 4 post-injection, and every 12 h from day 5 to day 8 post-infection		The phage-treated group showed a significant reduction in bacterial load, but there was no notable difference in the rate of wound healing.	(22)

^*a*^ CFU, colony-forming units; DFI, diabetic foot infection; MDR, multidrug resistant; MRSA, methicillin-resistant *S. aureus*; MSSA, methicillin-susceptible *S. aureus*; PFU, plaque-forming units.

## CLINICAL CASE REVIEW OF PHAGE THERAPY FOR *S. AUREUS*-RELATED DFIs

Building on the encouraging results observed in *in vivo* models, clinical case studies have further demonstrated the efficacy of phage therapy in patients with *S. aureus*-DFIs. Phage therapy is emerging as a promising approach in the management of these infections, especially for patients who do not respond adequately to conventional antibiotics. In these reports, phages were administered under compassionate use programs, often through temporary authorization frameworks, highlighting the experimental nature of these interventions. The data in these clinical case studies highlight a variety of patient experiences, underscoring the potential of phage therapy across diverse profiles, including variations in demographics, infection types, treatment approaches, and clinical outcomes (Table 2).

Phages showed significant outcomes when used alone in treating DFIs caused by *S. aureus* as reported by Fish et al. (30, 31). Seven diabetic patients with poorly perfused toe ulcers and exposed necrotic bone were treated weekly with the staphylococcal phage Sb-1. All patients responded positively, with ulcers healing within an average of 7 weeks, and one patient requiring up to 18 weeks. No adverse effects were observed, demonstrating the therapy's safety and effectiveness. Fish et al. (32) also documented that a 63-year-old female with a history of diabetic neuropathy and methicillin-susceptible *S. aureus* (MSSA)-infected ulcer on her distal right second toe was treated with local injections of the same phage Sb-1. Despite brief initial use of levofloxacin, which was discontinued due to ineffectiveness, the ulcer healed completely with re-ossification of the distal phalanx, and no recurrence of osteomyelitis after 3 years, further attesting to the efficacy of phage monotherapy (32).

On the other hand, phage cocktails demonstrated effectiveness in treating complex and chronic infections. Nadareishvili et al. (33) observed that four diabetic patients suffering from chronic osteomyelitis and other bacterial infections received daily treatments with a cocktail of Staphylococcal, Pyo, Intesti, and SES phages. This treatment led to significant improvement and healing with no adverse effects, highlighting the broad potential of phage cocktails in clinical settings (33).

The integration of phage therapy with antibiotics has also been explored, providing synergistic effects that enhance healing outcomes. Markoishvili et al. (34) noted that the use of PhagoBioDerm films impregnated with phages and ciprofloxacin in three patients resulted in complete ulcer closure. In one of these cases, the treatment was supplemented with clindamycin and metronidazole, demonstrating how combining phages with antibiotics can effectively manage complex infections (34). Patel et al. (35) reported that a large study involving 27 patients with chronic non-healing wounds treated with customized phage applications resulted in complete healing in 74% of the cases within 3 months. This study highlights the potential for customized phage therapy to adapt to specific clinical needs and bacterial profiles, offering a personalized approach to infection management. This flexibility and the ability to tailor phage treatments to the unique microbial environment of each patient infection underline the transformative potential of phage therapy (35, 36).

Young et al. (36) investigated the use of phage therapy in managing severe foot infections among nine diabetic patients at high risk of amputation (36). Patients received daily treatments with the staphylococcal ISP phage for durations ranging from 6 to 23 days. The therapy successfully resolved infections in five patients, highlighting its potential role in the treatment of advanced DFIs and in preventing limb loss (36).

However, while initial case studies and small clinical series suggest that phage therapy may be effective for treating *S. aureus*-related DFIs, particularly in antibiotic-resistant cases, it is important to note that these findings are also limited by several significant factors. First, the small sample sizes typical in these studies restrict the ability to generalize results and draw definitive conclusions about the therapy efficacy and safety. Additionally, the absence of control groups prevents establishing a clear causal relationship between the therapy and clinical outcomes. The variability in treatment protocols, including differences in phage preparations, dosages, and administration methods, complicates comparisons across studies and the development of standardized treatment guidelines. Furthermore, these studies are often subject to bias and subjectivity in reporting, with cases having favorable outcomes more likely to be published, and interpretations by researchers can vary. Lastly, the heterogeneity of patients in terms of medical history and infection severity affects treatment effectiveness and complicates the identification of optimal candidates for phage therapy.

**TABLE 2:** Summary of *S. aureus* DFIs treated with phage therapy^*a*^

Patient characteristics	Infection characteristics	Phage treatment approach	Combination therapy details	Treatment outcomes	Ref.
Seven patients (six males, one female), aged 44 to 92 years, with diabetes and multiple comorbidities (hypertension, hyperlipidemia, etc.)	Poorly perfused toe ulcers with exposed necrotic bone; MSSA, MRSA, and other staphylococcal infections; unsuccessful antibiotic treatments	Local application of staphylococcal phage Sb-1 weekly (10^7^–10^8^ PFU/mL)	N/A	Ulcers healed in 7 weeks on average, one case required 18 weeks; no observed adverse effects	(30, 31)
One female patient, 63 years old, with diabetes, diabetic neuropathy, hypertension, hyperlipidemia, and other disorders	Ulcer on distal right second toe with osteomyelitis, MSSA infection, unsuccessful topical antibiotic ointment	Local injection of staphylococcal phage Sb-1 into soft tissue and bone, weekly for 7 weeks	Levofloxacin 500 mg (briefly used without result and then discontinued)	Ulcer healed, re-ossification of distal phalanx, no recurrence of osteomyelitis after 3 years	(32)
Three patients (one male, two females), aged 50–69 years, with diabetes	Non-healing or recurrent foot ulcers, MSSA, *Staphylococcus epidermidis, P. aeruginosa, Streptococcus, Proteus*	Local application of PhagoBioDerm films impregnated with Pyophage preparation (*P. aeruginosa, E. coli, S. aureus, streptococcus* and *proteus* phages; 10^6^ PFU/cm^2^) and ciprofloxacin (0.6 mg/cm^2^), with film replacement every 3–7 days until healing	Clindamycin and metronidazole for one case, N/A for others	Complete closure in 12 days, 4 months, and 15 months	(34)
Two male patients, 60 years and not specified, with diabetes	DFUs, MRSA infections, unsuccessful antibiotic treatments	Local application of Pyo Bacteriophage or *Staphylococcus* phage preparation (10^7^–10^10^ PFU/mL) up to four times per day, for 2–3 weeks	N/A	MRSA eradication and significant improvement in ulcer healing in 3–4 weeks	(37)
Four patients (three males, one female) aged 60–74 years, with diabetes	Chronic osteomyelitis, DFUs, or post-operative infections with *S. aureus, Burkholderia cepacia, Enterococcus faecalis, Serratia marcescens*	Local and oral administration of Staphylococcal, Pyo, Intesti, and SES phage cocktails (> 10^7^ PFU/mL), daily	N/A	Significant improvement and healing, no adverse effects, follow-up ranging from 3 months to 2 years	(33)
Three patients (two males, one female), aged 56–67 years, with diabetes	Osteomyelitis and DFIs, *S. aureus* (MDR and susceptible)	Local application of cocktail TP-102 (*S. aureus, P. aeruginosa, A. baumannii* phages) from 6 to 45 days	Cefazolin and clindamycin for one case, N/A for others	Clinical and microbiological recovery in two cases and failure in one case	(38)
Twenty-seven patients with diabetes	Chronic non-healing wounds, *S. aureus, E. coli, P. aeruginosa, Klebsiella pneumoniae*	Local application of monophage or cocktail therapy, administered five to seven times on alternate days	N/A	20/27 patients (74%) achieved complete healing of chronic wounds within 3 months, with significant improvement in healing parameters; one patient exited the cohort during the follow-up period	(35)
Nine diabetic patients	Severe DFIs with high risk of amputation; MSSA*,* MRSA, coliforms, *Enterococcus faecalis, Corynebacterium amycolatum, E. coli, Corynebacterium striatum*	Local application of staphylococcal phage ISP (10^9^ PFU/mL), once daily from 6 to 23 days	Various intravenous antibiotics (flucloxacillin, gentamicin, daptomycin, cotrimoxazole, clindamycin, and piperacillin/tazobactam), administered either alone or in combination	Phage therapy resolved the infection and improved outcomes in eight patients but was unsuccessful in one.	(36)

^*a*^ DFIs, diabetic foot infections; DFUs, diabetic foot ulcers; MDR, multidrug resistant; MRSA, methicillin-resistant *S. aureus*; MSSA, methicillin-susceptible *S. aureus*; N/A, not applicable; PFU, plaque-forming units.

## CLINICAL TRIALS ASSESSING PHAGE THERAPY FOR *S. AUREUS*-RELATED DFIs

Clinical trials are crucial for evaluating the efficacy of phage therapy in treating *S. aureus*-related DFIs, providing key data through randomized, comparative, and blind designs. These studies are essential not only for confirming safety and effectiveness but also for establishing the evidence needed to potentially integrate phage therapy into existing DFI treatment guidelines. Table 3 presents a summary of ongoing trials, including REVERSE, REVERSE2, DANCE, and PhagoPied, each assessing various aspects of phage therapy and evaluation parameters such as wound healing, microbial reduction, adverse events, and pathogen eradication.

One notable aspect of these trials is their focus on rigorously evaluating the safety and efficacy of phage therapy in clinical settings. The REVERSE trial, for instance, is investigating microbial reduction and wound healing improvements in patients with infected DFIs treated with the TP-102 phage cocktail. According to recently published results, TP-102 was found to be safe and well tolerated, with no treatment-related adverse events reported during the study. A greater proportion of patients in the TP-102 cohort demonstrated microbial reduction and wound closure (50%–70%) compared to the placebo group, and one patient achieved complete wound closure, underscoring the potential of TP-102 to promote healing in challenging infections. However, the trial's small sample size limited its statistical reliability to draw definitive conclusions (39). To address these limitations, the ongoing REVERSE2 trial is enrolling a larger cohort, focusing on more severe diabetic foot ulcers infected with *P. aeruginosa*, *S. aureus*, or *Acinetobacter baumannii*. REVERSE2 aims to collect data on adverse events, wound reduction, inflammatory markers, and pathogen eradication, potentially validating and expanding upon the findings of the REVERSE trial.

Additionally, the DANCE trial stands out for its use of personalized phage therapy, where treatment is tailored based on phage susceptibility testing. This approach may enhance specificity and effectiveness against *S. aureus*, as it ensures that the phages selected are optimally suited to target the strains present in each patient infection profile. Meanwhile, the PhagoPied trial evaluates a standardized anti-*Staphylococcus* phage application regimen and interestingly monitors the development of anti-phage antibodies, which introduces an additional layer of complexity, as the immune response may influence phage efficacy over time. This parameter, which had not been extensively measured in previous studies, provides valuable insight into the immune dynamics affecting phage therapy.

However, these trials also highlight certain limitations and challenges associated with phage therapy. Many are still in early stages, such as Phase I or Phase II, with sample sizes typically ranging from 20 to 120 participants. While this is consistent with early-phase antibiotic trials, such small cohorts limit the generalizability of results and preclude definitive conclusions about efficacy and safety. Larger Phase III studies, involving several hundred participants, will be necessary to robustly confirm the clinical benefits of phage therapy.

**TABLE 3:** Summary of ongoing clinical trials assessing phage therapy for *S. aureus* DFIs^*a*^

Trial	Study group	Treatment group	Placebo group	Evaluation parameters	Ref.
“REVERSE” Phase I/IIa, 20 participants	DFUs non-infected or infected with *P. aeruginosa*, *S. aureus,* and/or *A. baumannii*	Topical application of phage treatment (cocktail TP-102 targeting *S. aureus*, *P. aeruginosa*, and *A. baumannii*; 10⁹ PFU/mL), administered three times per week for 1 week in uninfected ulcers and 4 weeks in infected ulcers	Topical application of a placebo following the same schedule as the treatment group	Frequency and severity of expected and unexpected adverse events, microbial reduction or eradication, and wound healing	Technophage (Portugal) clinicaltrials.gov #NCT04803708 (39)
“REVERSE2” Phase IIb, 80 estimated participants	Severe DFUs infected with *P. aeruginosa*, *S. aureus* or *A. baumannii*	Topical application of phage treatment (cocktail TP-102 targeting *S. aureus*, *P. aeruginosa*, and *A. baumannii*; 10⁹ PFU/mL), administered three times per week for 4 weeks	Topical application of a placebo following the same schedule as the treatment group	Adverse events, wound size reduction, inflammatory marker changes, pathogen eradication, and resistance emergence	Technophage (Portugal) clinicaltrials.gov #NCT05948592
“DANCE” Phase IIa, 126 estimated participants	Diabetic foot osteomyelitis infected with *S. aureus*	Personalized phage therapy based on phage susceptibility testing	Saline solution administered following the same schedule and techniques as the phage therapy group	Reduction in ulcer area and healing, C-reactive protein decrease, pathogen eradication, and occurrence of adverse events	Adaptive Phage Therapeutics (United States) clinicaltrials.gov #NCT05177107
“PhagoPied” Phase I/II, 60 estimated participants	DFUs infected with MRSA or MSSA	Topical application of anti-*Staphylococcus* bacteriophage (10⁷ PFU/mL), administered on days 0, 7, and 14 of the treatment period	Topical application of a placebo following the same schedule as the treatment group	Wound depth and area measurements, immediate safety assessment, healing time and rate, pathogen identification, wound microbiota analysis, and detection of anti-phage antibodies	Centre Hospitalier Universitaire de Nîmes (France) clinicaltrials.gov #NCT02664740

^*a*^ DFUs, diabetic foot ulcers; MRSA, methicillin-resistant *S. aureus*; MSSA, methicillin-susceptible *S. aureus.*

## GENERAL CONCLUSION

This review provides a comprehensive overview of the potential of phage therapy for *S. aureus*-related DFIs, covering the strengths and limitations of various animal models and highlighting the need for Type 2 diabetes models to more accurately reflect the diabetic profile most prevalent in DFI patients. Clinical case studies further show promising outcomes in infection control and wound healing, especially in antibiotic-resistant cases, while emerging clinical trials could provide rigorous evaluations of phage safety and efficacy. Beyond DFIs, phage therapy is increasingly being explored for other difficult-to-treat infections, including respiratory, urinary tract, and bone and joint infections. These efforts often target pathogens such as *P. aeruginosa*, *Escherichia coli*, and *S. aureus*, highlighting the broader clinical relevance of phages. However, several major challenges remain. These include the need for harmonized regulatory frameworks, the current reliance on compassionate use, the complexity and cost of producing pharmaceutical-grade phages, and the necessity to anticipate and manage bacterial resistance. Concrete efforts are underway to address these issues. Regulatory discussions are advancing at the European level to support personalized phage therapies. At the same time, initiatives such as dynamic phage banks, real-time adaptation of phages, and phage-antibiotic combination strategies are being developed to improve outcomes and limit resistance. Altogether, these findings emphasize the growing therapeutic potential of phage therapy in complex infections and the need for further research to support its integration into standardized clinical practice.
